# Nuclear Focal Adhesion Kinase Protects against Cisplatin Stress in Ovarian Carcinoma

**DOI:** 10.1158/2767-9764.CRC-24-0382

**Published:** 2024-12-20

**Authors:** Yichi Zhang, Marjaana Ojalill, Antonia Boyer, Xiao Lei Chen, Elise Tahon, Gaëtan Thivolle Lioux, Marvin Xia, Maryam Abbas, Halime Meryem Soylu, Douglas B. Flieder, Denise C. Connolly, Alfredo A. Molinolo, Michael T. McHale, Dwayne G. Stupack, David D. Schlaepfer

**Affiliations:** 1 Division of Gynecologic Oncology, Department of Obstetrics, Gynecology, and Reproductive Sciences, Moores Cancer Center, University of California, San Diego, La Jolla, California.; 2 Fox Chase Cancer Center, Philadelphia, Pennsylvania.; 3 Department of Pathology, Moores Cancer Center, University of California San Diego, La Jolla, California.

## Abstract

Tumor chemotherapy resistance arises frequently and limits high-grade serous ovarian cancer (HGSOC) patient survival. Focal adhesion kinase (FAK) is an intracellular protein–tyrosine kinase encoded by *PTK2*, a gene that is often gained in HGSOC. Canonically, FAK functions at the cell periphery. However, FAK also transits to the nucleus to modulate gene expression. We find that FAK is tyrosine-phosphorylated and nuclear-localized in tumors of patients with HGSOC surviving neoadjuvant platinum–paclitaxel chemotherapy and that FAK nuclear accumulation occurs upon subcytotoxic cisplatin exposure to ovarian tumor cells *in vitro*. FAK nuclear localization sequence (NLS) mutational inactivation resulted in tumor cell sensitization to cisplatin *in vitro* and *in vivo* relative to wild-type FAK-reconstituted ovarian tumor cells. Cisplatin cytotoxicity was associated with elevated ERK MAPK activation in FAK NLS^−^ cells, cisplatin-stimulated ERK activation was also enhanced upon loss of FAK activity or expression, and cisplatin-stimulated cell death was prevented by an inhibitor of ERK signaling. MAPK phosphastase-1 (MKP1) negatively regulates ERK signaling, and cisplatin-induced MKP1 levels were significantly elevated in wild-type FAK compared with FAK NLS^−^ ovarian tumor cells. Notably, small-molecule MKP1 inhibition enhanced both cisplatin-stimulated ERK phosphorylation and ovarian tumor cell death. Together, our results show that FAK expression, activity, and nuclear localization limit cisplatin cytotoxicity in part by regulating MKP1 levels and preventing noncanonical ERK/MAPK activation.

**Significance:**

FAK inhibitors are in combinatorial clinical testing with agents that prevent Ras–Raf–MAPK pathway activation in various cancers. This study suggests that nuclear FAK limits ERK/MAPK activation in supporting HGSOC cell survival to cisplatin stress. Overall, it is likely that targets of FAK-mediated survival signaling may be tumor type– and context-dependent.

## Introduction

High-grade serous ovarian carcinoma (HGSOC) is the deadliest gynecologic cancer in the United States (1). Standard-of-care treatment for HGSOC involves cytoreductive surgery followed by cisplatin/carboplatin (DNA damage generation) with paclitaxel (microtubule-stabilizing drug) chemotherapy (2). Although most patients initially respond to primary chemotherapy (3), approximately 80% of patients with HGSOC will exhibit tumor recurrence within 5 years, will progressively develop chemotherapy resistance after retreatment, and will eventually succumb to this disease (4).

Focal adhesion kinase (FAK) is an intracellular protein–tyrosine kinase product of the *PTK2* gene located at chromosome 8q24.3 (5) that undergoes gains and amplification in breast, uterine, cervical, and ovarian tumors (6). In HGSOC, elevated FAK mRNA and protein levels are associated with tumor progression and a poor prognosis (7). Whereas FAK is canonically known for promoting cell movement in coordination with integrin receptors at the cell periphery (8), the N-terminal FAK band 4.1, ezrin, radixin, and moesin (FERM) domain can mediate FAK nuclear localization (9, 10).

In the nucleus, FAK modulates gene expression in both kinase-dependent and -independent manners (11, 12). IHC staining of tumors with antibodies to the FAK tyrosine-397 (pY397) autophosphorylation site revealed general increased stromal FAK pY397 staining in pancreatic carcinomas (13), whereas in breast carcinomas, nuclear FAK pY397 staining was detected in both tumor and associated blood vessel endothelial cells (13, 14). Although nuclear FAK IHC staining is associated with a poor prognosis in human colorectal cancer (15) and FAK signaling supports tumor chemotherapy resistance (16), it remains unclear whether FAK nuclear localization contributes to HGSOC cisplatin resistance.

The ERK MAPK signaling pathway regulates diverse cellular processes such as cell proliferation, cell motility, and gene expression (17). ERK activation is generally associated with the growth and survival of many cancers. To this end, FAK phosphorylation at Y925 facilitates Grb2 adapter protein binding and creates a signaling linkage to ERK activation downstream of integrins (18). Despite numerous findings of ERK activation promoting tumorigenesis (19), there is evidence that noncanonical ERK activation can be also connected to the enhanced sensitivity of tumor cells to cisplatin-induced cell death (20, 21). MAPK phosphatase-1 (MKP1, also known as DUSP1) negatively regulates ERK activity, and MKP1 mRNA and protein levels are increased in response to cisplatin (21). Interestingly, MKP1 expression is elevated in HGSOC, MKP1 is associated with cisplatin resistance, and small-molecule inhibitors of MKP1 phosphatase activity inhibit HGSOC cell proliferation and promote cell death (22–24). These findings support a complex noncanonical signaling relationship between MKP1 and ERK in mediating cisplatin cytotoxicity, impacting HGSOC cell survival.

Herein, we find that FAK is tyrosine-phosphorylated and localized to the nucleus of patients’ HGSOC tumor cells after neoadjuvant platinum and paclitaxel chemotherapy. In human and murine ovarian carcinoma cells, subcytotoxic cisplatin stress facilitates FAK nuclear accumulation and is associated with elevated MKP1 levels. Inhibition of MEK1 upstream of ERK was cisplatin-protective, whereas small-molecule inhibitors of MKP1 enhanced cisplatin-induced cell death. Paradoxically, loss of FAK expression or activity was associated with elevated ERK activation after cisplatin stimulation. As FAK inhibition is in clinical testing for Kirsten rat sarcoma virus (KRAS) oncogene-mutated non–small cell lung cancer (NSCLC; ref. 25) and low-grade serous ovarian cancer (LGSOC; ref. 26) as a therapy targeting “resistance” pathways activated by Ras/RAF/MEK pathway inhibition, our results linking FAK to the prevention of ERK activation via elevated MKP1 phosphatase expression in response to cisplatin stress may be associated with an alternate survival pathway in HGSOC. Taken together, our results support the notion that FAK effects on downstream signaling pathways may be tumor type– and chemotherapy-dependent.

## Materials and Methods

### Cell lines and cultures

Murine ovarian KMF cells were isolated and cultured as described (27), and OVCAR3 (RRID: CVCL_0465) cells were obtained from the ATCC (HTB-161). KMF FAK knockout (KO; clone KT13) and OVCAR3 FAK KO (clone AB21) were isolated and characterized as described (28). OVCAR4 cells (RRID: CVCL_1627) were obtained from the NCI by Materials Transfer Agreement (MTA) Division of Cancer Treatment and Diagnosis Tumor Repository and cultured as recommended. Initial validated cell lines were expanded, stored frozen, and used within 15 passages incorporating routine morphology checks. Passage 1 patient-derived xenograft (PDX) OC49 ovarian serous cystadenocarcinoma cells from paracentesis were provided by Fox Chase Cancer Center by MTA. Briefly, early-passage (<6) OC49 cells were plated on mitomycin C pretreated 3T3-J2 fibroblasts grown in iron-supplemented calf serum (HyClone, SH30072), expanded to a 10-cm dish, replated on tissue culture–treated dishes, and used for experimental analyses at 48 hours. PDX Standard Media consists of 60% Ham’s F-12 media (Gibco, 11765054), 35% DMEM (Gibco, 11995073), 5% FBS (R&D Systems, S12450), 0.4 μg/mL hydrocortisone (Stem Cell Technologies, 07904), 5 μg/mL insulin (Gibco, 12585014), 8.4 ng/mL cholera toxin (List Biological Laboratories Inc., 100B), 10 ng/mL EGF (Gibco, PHG0313), 24 μg/mL adenine (Alfa Aesar, A14906), 10 μmol/L ROCK inhibitor (Tocris, Y-27632), 1× penicillin–streptomycin (Gibco, 15140122), and 1× L-glutamine (Gibco, 25030081). Cells were checked every 6 months using MycoScope PCR Detection Kit (Genlantis, MY01100).

N-terminal-tagged GFP-FAK and GFP-FAK R177A R178A [nuclear localiztion sequence (NLS^−^); murine cDNAs] were cloned into EF1A-promoter lentiviral vector (VectorBuilder Inc.). KT13 KMF or AB21 OVCAR3 cells were transduced with 100 multiplicity of infection recombinant lentivirus, and cells were sorted by flow cytometry for stable GFP expression using standard methods (28). Hygromycin B (200 μg/mL, 2 days) addition was used to maintain GFP-FAK expression. For labeling KMF cells for *in vivo* tumor studies, cells were transduced with pUltra-Chili-Luc (gift from Malcolm Moore (Addgene, plasmid # 48688) for bicistronic expression of dTomato and luciferase- and flow cytometry–sorted for equivalent dTomato and GFP expression between FAK wild-type (FAK-WT) and FAK-NLS^−^ cells prior to initiating tumor experiments in mice.

### Antibodies/reagents

Cisplatin (Westward Pharmaceuticals, 0143950401), paclitaxel (TEVA Pharmaceuticals, 1384921), Matrigel (Corning, 356231), BCI-215 (MedChemExpress, HY-121087), FAK PROTAC-1 (MedChemExpress, HY-119932), and FAK PROTAC FC-11 (Tocris, 7306) were purchased. FAK inhibitor (FAKi; IN10018) was otained from InxMed Inc by MTA. Antibodies to β-tubulin (2146S, RRID: AB_2210545), caspase 3 (9662S, RRID: AB_331439), active-ERK phospho- (pERK1/2; pT202/pY204; 9101S, RRID: AB_331646), total ERK (Erk1/2; 9102S, RRID: AB_330744), active p38 MAPK (pT180/pY182; 9211S, RRID: AB_331641), p38 MAPK (9212S, RRID: AB_330713), active-JNK phospho- (JNK1/2; pT183/pY185; 9255, RRID: AB_2307321), total JNK (E7R5D, RRID: AB_2141027), histone-3 (4499S, RRID: AB_10544537), DUSP-1/MKP1 (clone E6T5S, RRID: AB_3371713), DUSP1/MKP1 (35217, RRID: AB_2094225), and antibodies to Pyk2 (clone 5E2, 3480S, RRID: AB_2174093) were from Cell Signaling Technology. Mouse mAb to FAK (clone 4.47, 05-537, RRID: AB_2173817) was from Millipore Sigma. Anti–FAK pY576 (ab226847, RRID: AB_880072) and antibodies to FAK pY397 (clone EP2160Y, RRID: AB_448317) were from Abcam.

### Patient tumor samples and IHC staining

De-identified human ovarian cancer tissue specimens from consented patients were obtained from Fox Chase Cancer Center (FCCC) Biosample Repository Facility (BRF) under Institutional Review Board (IRB)-approved protocols (IRB 11–866 and IRB 08–851). FCCC staff queried the BRF sample database to identify participants who received carboplatin and paclitaxel neoadjuvant chemotherapy. Biopsy specimens were obtained from FCCC Surgical Pathology, sectioned, hematoxylin and eosin–stained and reviewed by a board-certified pathologist. Formalin-fixed, paraffin-embedded blocks from the biopsy and the corresponding surgical resection blocks banked by the BRF were cut to obtain one hematoxylin and eosin–stained slide and additional unstained sections. One section each from pretreatment biopsy and postneoadjuvant treatment surgical resection specimen was stained for Pax8 by FCCC Histopathology Facility to confirm tumor presence (28). The remaining unstained slides were sent to the University of California San Diego (UCSD) for additional staining performed under UCSD IRB-approved protocol (IRB 110805). Patient tumor samples 1013983, 1014252, 1016247, 1014109, 1014386, and 3000276 were also previously analyzed for FAK pY397 IHC staining (28).

Patient pretreatment biopsy and postneoadjuvant chemotherapy surgical resection tumor tissue samples were stained for FAK pY576 reactivity. Briefly, tissue sections (5 μm) were baked at 60°C for 1 hour, cleared, and rehydrated through successive alcohols (3 × xylene, 2% × 100% EtOH, 2% × 95% EtOH, and 2% × 70% EtOH) and then water. Antigen retrieval was performed using Antigen Unmasking Solution (Tris-based, pH 9; VectorLabs, H3301) at 95°C for 30 minutes. Staining was performed using an intelliPATH automated IHC stainer (Biocare) with the following parameters: peroxidase block Bloxall (VectorLabs, SP-6000) for 10 minutes; protein block Rodent Block M (Biocare, RBM961H) for 1 hour, or 3% donkey serum for 10 minutes. Primary FAK pY576 antibody (1:1,000) incubation was carried out for 1 hour. Secondary antibody incubation with anti-rabbit HRP polymer (Cell IDX, 2RH-100) was carried out for 30 minutes. 3, 3′-Diaminobenzidine (brown) chromogen (VWR, 95041-478) staining was carried out for 5 minutes. Slides were counterstained with Mayer’s hematoxylin (Sigma-Aldrich, 51275) for 5 minutes, dehydrated, cleared, and coverslip-mounted. Normal and human tumor tissues were used as controls to optimize staining. Rabbit anti–FAK pY576 did not stain FAK-null murine ovarian tumors (28). Digital color scans were acquired (Aperio AT2 scanner) using ImageScope software (Leica Biosystems). Images were also acquired using an upright microscope (Olympus BX43) with a color camera (Olympus SC100). Quantitative image analysis was performed using QuPath v0.4.2 software (29). Briefly, images were annotated, FAK pY576–positive cells were identified by creating a composite classifier, and 3, 3′-diaminobenzidine nuclei were identified using cell detection command.

### Immunoblotting

Cells in culture or collected by centrifugation of peritoneal-associated cells from tumor-bearing mice were washed with cold PBS, whole-cell protein lysates were made by RIPA Lysis or Extraction Buffer (Pierce, 89900) addition, and lysates were clarified by centrifugation (16,000 × *g*, 5 minutes). Non-denaturing partitioning of proteins into nuclear and cytoplasmic fractions was performed using NE-PER Nuclear and Cytoplasmic Extraction Reagents (Thermo Fisher Scientific, #78835) following the manufacture’s recommendations. Complete Mini ETDA-free Protease inhibitor cocktail (Millipore Sigma, 11836170001) and PhoSTOP phosphatase inhibitor cocktail (Millipore Sigma, 4906845001) were added to lysis buffer prior to use. Total protein levels were determined using a bicinchoninic acid assay (Pierce), 25 μg of protein were resolved on Mini-Protean TGX Precast Gel (4%–15% Tris/Glycine gel, Bio-Rad, 456-1086), and transferred to polyvinylidene difluoride (PDVF) membranes (Bio-Rad) using a TransBlot Turbo (Bio-Rad) for subsequent immunoblotting. Immunoreactive protein bands were detected using HRP-conjugated anti-mouse or anti-rabbit antibodies with Clarity Western ECL (enhanced chemiluminescence) substrate (Bio-Rad, 1705061) reagent and visualized using ChemiDoc Touch Imaging System (Bio-Rad). Where indicated, protein chemiluminescence signal was quantified using Image Lab software (v6.1, Bio-Rad, RRID: SCR_014210) with automatic lane and band identification.

### Cisplatin and paclitaxel cytotoxicity

Cells were seeded (1 × 10^4^ cells per well) in 96-well plates (Corning), and after 24 hours, increasing concentrations of cisplatin (made by serial dilutions), paclitaxel, or DMSO (control) were added to wells. After 48 hours, alamarBlue Reagent (Invitrogen, 2706294) was added, cells were incubated at 37°C in 5% CO_2_ for 4 hours, and 570/600 nm light absorbance was measured using a plate reader (SpectraMax ID3, Molecular Devices). Mean values were determined from triplicate points, relative cell viability was calculated versus DMSO control, and at least three independent experiments were performed. IC_50_ values were determined using Prism (v10, RRID: SCR_005375) from GaphPad software.

### Colony formation assays

Cells were seeded (6,000 cells/well) in 6-well plates (Corning), and after 24 hours, the indicated cisplatin concentration or DMSO (control) was added. After 10 days, cells were washed with PBS, fixed with ethanol, and then stained with 1% crystal violet dye for 15 minutes at room temperature (RT). Plates were washed with water, allowed to air dry overnight, and photographed. For quantitation, dye was eluted with methanol addition (20 minutes with rocking), and the optical absorbance of each well was measured at 570 nm using a plate reader. Mean values were determined from triplicate points, crystal violet signal obtained from wells with no cells was subtracted from all points, and colony formation was calculated as a percent of DMSO control. At least two independent experiments were performed.

### Cisplatin cell signaling

Equivalent number of cells were plated in growth media, and after 24 hours, the indicated concentration of cisplatin was added with or without MEK1 (U0126, 10 μmol/L), MKP1 (BCI-215, 1 μmol/L) inhibitor, or DMSO (control) addition, and cell lysates were analyzed at 1, 12, 24, or 48 hours. In the indicated experiments, cells were plated in the presence of FAKi (InxMed, IN10018, 1 μmol/L), FAK-specific (FAK PROTAC-1, 1 μmol/L), or FAK-Pyk2 targeting (FAK PROTAC FC-11, 1 μmol/L) in growth media for 48 hours and, media was changed with cisplatin addition for 24 hours with repeat addition of FAKi or FAK PROTAC prior to cell lysis with RIPA buffer.

### Mouse orthotopic tumor growth

All animal experiments were performed in accordance with The Association for Assessment and Accreditation for Laboratory Animal Care guidelines and approved by the UCSD Institutional Animal Care and Use Committee (protocol S07331). For KMF FAK-WT or FAK-NLS^−^ tumor growth and ascites analyses, 7.5 million pChili-luciferase–labeled cells in 100 μL DMEM were suspended with 100 μL phenol red–free high concentration of Matrigel (Corning, 356231) and i.p. injected into 8-week-old female C57Bl6/N mice (Charles River Laboratories). Tumor growth was monitored via bioluminescent luciferase imaging at indicated days (IVIS, PerkinElmer), mice were randomized at day 5, and mice received i.p. injections of cisplatin (2 or 4 mg/kg) or saline (control) every 7 days.

Peritoneal ascitic tumor plus immune cells were harvested by injection of 5 mL collection buffer (PBS containing 2 mmol/L EDTA and 2% BSA). Volume of up to 10 mL was collected, red blood cells were lysed (RBC lysis buffer, eBioscience, 00-4300-54) for 5 minutes at RT, reactions were stopped by dilution with collection buffer at 4°C, and cells were pelleted by centrifugation (2,000 rpm for 6 minutes at 4°C). Cell pellets were resuspended in 2 mL PBS containing 4 mmol/L EDTA and 2% BSA and mechanically dissociated by gentle and repeated pipetting (1,000-μL tip). Cells were diluted by the addition of PBS with 2 mmol/L EDTA and 2% BSA, filtered through a 70-μm cell strainer, and enumerated as a single-cell suspension with trypan blue dye exclusion analyses (ViCell XR).

### Immunofluorescence analyses

GFP-FAK localization was visualized by plating KMF cells on glass coverslips coated with 0.1% gelatin, and after 24 hours, media was changed to contain DMSO or cisplatin (20 μmol/L) for 12 or 24 hours. Coverslips were washed with PBS, fixed in 3.7% paraformaldehyde (Electron Microscopy Sciences, 15710-S) for 10 minutes at RT, permeabilized with 0.1% Triton X-100, and blocked with 2% BSA in PBS for 1 hour at RT. Cell nuclei were counterstained with 4′,6-diamidino-2-phenylindole (DAPI, 1 μg/mL) in blocking buffer for 10 minutes, mounted using VECTASHIELD PLUS Antifade Mounting Medium (Vector Laboratories, H-1900) onto glass slides, and sealed with nail polish. Images of GFP-FAK and nuclei were acquired using a Nikon A1R confocal microscope, automated XYZ stage, Plan Apo 40× oil objective, LU4 four-laser acousto-optic tunable filter unit with 405-, 488-, 561-, and 647-nm lasers, an Andor iXon Ultra 897 EMCCD camera, and NIS-Elements Imaging Software. Where indicated, cells were also treated with leptomycin B (50 nmol/L) for 6 hours, fixed in 3.7% paraformaldehyde, and visualized. FAK-WT and FAK-NLS^−^ KMF cell death in the presence 20 μmol/L cisplatin for 48 hours was measured by terminal deoxynucleotidyl transferase–mediated dUTP nick end labeling (TUNEL) staining using the TMR red kit (Roche) according to manufacture directions. Cells were stained with DAPI with green (GFP-FAK), red (TUNEL), and blue (DAPI) fluorescence images acquired using a Nikon A1R confocal microscope as above. Ten TUNEL-stained images from three coverslips were acquired. Only cells with TUNEL-positive staining colocalized with DAPI were counted.

### Kaplan–Meier

mRNA expression array data were evaluated using the Kaplan–Meier plotter (www.kmplot.com/ovar). The MKP1 probe (Affy ID: 201041_s_at) was used with query parameters of progression-free survival and auto-select best cutoff. Expression range of probe was 17-24322, and the cutoff value used in analysis was 3,535. Restrictions were serous histology, stages (2+3+4), all grades, TP53 mutation, optimal surgical debulking, and received platin chemotherapy, and all available datasets were used. A total of 949 patient samples were analyzed.

### Statistics

Analyses were performed in Prism v10 (GraphPad Software). For experimental groups of three or more, statistical significance was calculated based on one-way ANOVA with Tukey multiple comparison test. Unpaired *t* test was used to determine statistical difference between the means from two different samples. *P* values < 0.05 were considered significant.

### Data availability

The data generated in this study are available upon request from the corresponding author.

## Results

### Active FAK staining increases in the nucleus of HGSOC patient tumor cells surviving neoadjuvant chemotherapy

**Figure 1:**
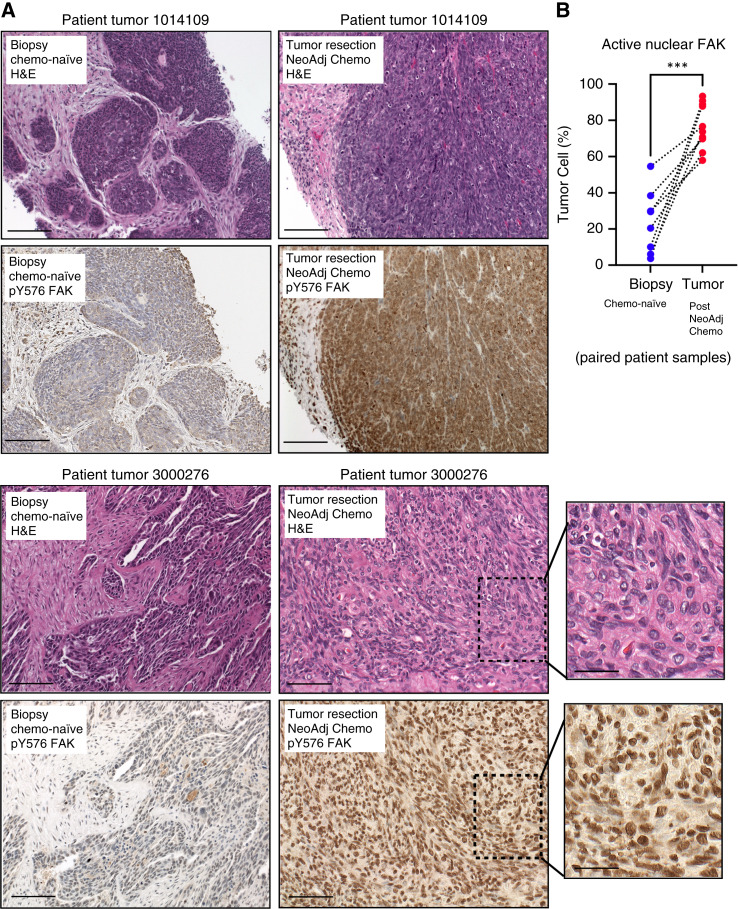
Active FAK staining increases in the nucleus of HGSOC patient tumors after neoadjuvant chemotherapy. **A,** IHC staining of paraffin-embedded serial initial tumor biopsy sections (patient 1014109 and patient 3000276) and corresponding tumor resection after neoadjuvant chemotherapy treatment. Samples were stained with H&E and phosphospecific antibodies to the FAK activation loop Y576 (pY576) within the kinase domain. Scale is 100 μm (inset scale is 25 μm). **B,** Image quantification of percent of tumor cell area exhibiting FAK pY576 staining. Paired tumor samples (*n* = 8) from initial biopsy (blue circles) and from surgical tumor debulking after neoadjuvant chemotherapy (red circles). Dotted lines connect paired patient tumor samples collected prior to and after neoadjuvant chemotherapy (***, *P* < 0.001). Chemo, chemotherapy; H&E, hematoxylin and eosin; NeoAdj, neoadjuvant.

FAK can be activated by stepwise phosphorylation events initiated at Y397 (pY397) that culminate in phosphorylation at FAK Y576 (pY576) within the kinase domain and full catalytic activation (5, 30). Thus, phosphospecific antibodies to FAK pY397 or FAK pY576 can serve as indirect reporters of FAK activation (31). Previously, we showed that FAK pY397 IHC staining of HGSOC patient samples increased in tumors surgically removed after three or more rounds of neoadjuvant carboplatin–paclitaxel chemotherapy compared with initial biopsy specimens from the same patients (28). As FAK pY397 staining was present throughout tumor cells, quantifying changes in FAK protein localization using this antibody was not possible. Herein, using some of the same paired patient tumor samples (28), only weak and diffuse FAK pY576 staining was detected in tumors collected at initial biopsy (chemo-naïve; Fig. 1A). In contrast, strong FAK pY576 staining was detected in tumor specimens after chemotherapy and FAK pY576 staining was highly nuclear-localized (Fig. 1A). Quantitative image analyses revealed that nuclear FAK pY576 trended significantly upward after neoadjuvant chemotherapy in the paired patient samples (Fig. 1B). These results support a potential association between active nuclear FAK and HGSOC patient tumor cells surviving neoadjuvant chemotherapy.

### Nuclear FAK accumulation occurs upon cisplatin treatment of ovarian tumor cells

**Figure 2:**
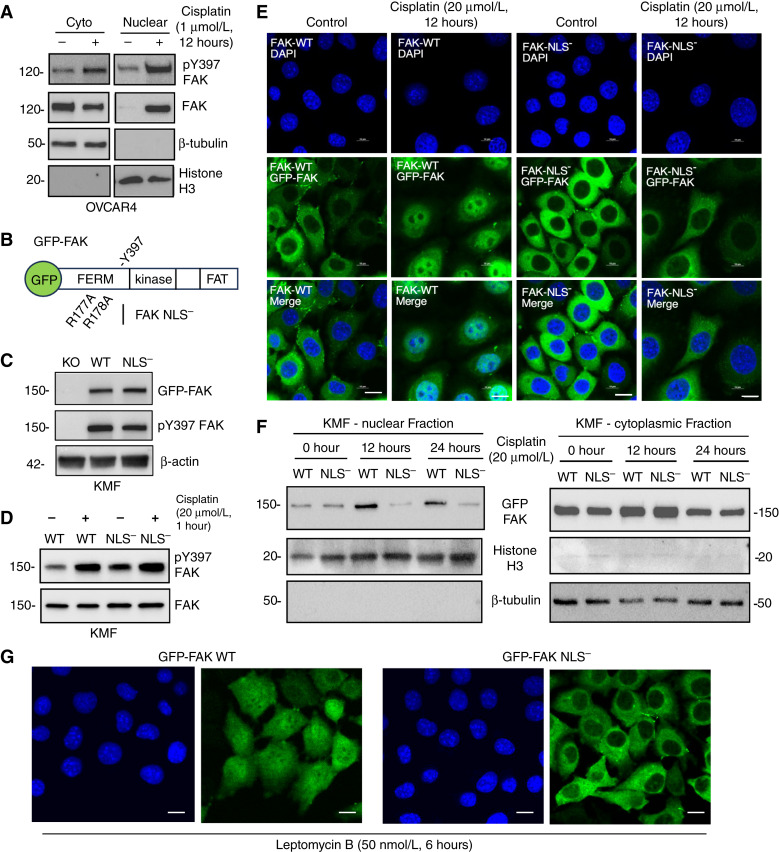
Nuclear FAK accumulation occurs upon cisplatin treatment of human and murine ovarian tumor cells. **A,** Nuclear and cytoplasmic cell fractionation of human OVCAR4 cells treated with DMSO (control) or cisplatin (1 μmol/L) for 12 hours followed by immunoblotting for FAK pY397, total FAK, β-tubulin (cytoplasmic marker), and histone H3 (nuclear marker). **B,** Schematic of GFP fusion to FAK denoting N-terminal band 4.1, ezrin, radixin, moesin homology (FERM), kinase, and focal adhesion targeting (FAT) domains. FAK FERM arginine R177 and R178 to alanine mutational changes prevent FAK nuclear localization. **C,** Stable reexpression of GFP-FAK-WT (WT) and GFP-FAK-NLS^−^ (NLS^−^) in KMF FAK KO murine ovarian tumor cells as analyzed by FAK, pY397 FAK, and β-actin immunoblotting. **D,** KMF FAK-WT and KMF FAK-NLS^−^ cells treated with DMSO (control) or cisplatin (20 μmol/L) for 1 hour and immunoblotting for FAK pY397 and total FAK. **E,** Confocal immunofluorescence imaging was used to visualize GFP-FAK and DAPI stain in FAK-WT and FAK-NLS^−^ cells treated with DMSO (control) or cisplatin (20 μmol/L) for 12 hours. Shown are representative images of GFP-FAK (images shown at midline of Z-stack), nucleus, and merged images. Scale bar, 10 μm. **F,** Nuclear and cytoplasmic fractionation of FAK-WT and FAK-NLS^−^ cells treated with cisplatin (20 μmol/L) for 0, 12, or 24 hours followed by immunoblotting for FAK, β-tubulin, and histone H3. **G,** Representative images of GFP-FAK-WT and FAK-NLS^−^ expressing (images shown at midline of Z-stack) and DAPI-stained cells treated with leptomycin B (50 nmol/L) for 6 hours. Scale bar, 10 μm. Cyto, cytoplasmic.

We previously showed that FAK pY397 phosphorylation is increased by exposure of human OVCAR3 and murine KMF ovarian tumor cells to subcytotoxic concentrations of cisplatin in culture (28). To determine if endogenous FAK exhibits nuclear accumulation upon cisplatin treatment, human OVCAR4 HGSOC cells were treated with 1 μmol/L cisplatin (below the determined IC_50_ value) or DMSO (control), and protein lysates were biochemically separated into cytoplasmic- and nuclear-enriched fractions and then immunoblotted for total and FAK pY397 (Fig. 2A). Under control conditions, the majority of OVCAR4 FAK is cytoplasmic localized, whereas 12 hours after cisplatin addition, high levels of Y397 phosphorylated FAK were detected in the nuclear-enriched cell fraction.

Previous studies have identified a basic amino acid–rich NLS motif within the FAK FERM domain in which mutation of arginine (R)-177 and R-178 to alanine (A; R177A R178A, NLS^−^) can limit FAK nuclear localization (Fig. 2B; ref. 32). Using a CRISPR-generated and fully sequenced FAK KO clone (KT13) of KMF cells, GFP fusion proteins of FAK-WT or FAK-NLS^−^ were stably and equally reexpressed in KMF FAK-KO cells (Fig. 2C). In growing cell cultures, FAK-WT and FAK-NLS^−^ were phosphorylated at Y397, and this increased within 1 hour after 20 μmol/L cisplatin addition (Fig. 2D). Previously, the cisplatin IC_50_ value of parental KMF cells was determined to be 31 μmol/L as these cells are considered cisplatin-resistant (28). Herein, experimental conditions were performed at concentrations below cellular IC_50_ values for KMF cisplatin cytotoxicity.

To visualize whether cisplatin addition results in FAK nuclear accumulation in intact cells, GFP-FAK distribution was analyzed by confocal microscopy in FAK-WT and FAK-NLS^−^ KMF cells in growth media (control) or in media with cisplatin (20 μmol/L, 12 hours; Fig. 2E). Shown are DAPI-stained (nuclear), representative Z-plane stacked images for GFP-FAK, and a merged composite image (Fig. 2E). FAK-WT and FAK-NLS^−^ exhibited equivalent cytoplasmic distribution in control conditions, and only FAK-WT showed nuclear accumulation after cisplatin addition. Biochemical fractionation of cisplatin-treated FAK-WT and FAK-NLS^−^ cell lysates into nuclear and cytoplasmic fractions followed by immunoblotting confirmed increased FAK-WT nuclear accumulation within 12 hours and that FAK-WT remained nuclear localized at 24 hours in cisplatin-treated cells (Fig. 2F). FAK-NLS^−^ remained in the cytoplasm fraction at 12 and 24 hours. As addition of the nuclear export inhibitor leptomycin B resulted in GFP-FAK-WT but not FAK-NLS^−^ nuclear accumulation within 6 hours (Fig. 2G), we conclude that FAK possesses nuclear function(s) in growing cells and that cisplatin stress likely alters the equilibrium of FAK cytoplasmic and nuclear distribution. Importantly, as FAK-NLS^−^ exhibits cisplatin-stimulated Y397 phosphorylation but not nuclear localization, our results support the notion that initial cisplatin-initiated FAK activation events are not necessarily linked to FAK nuclear accumulation.

### FAK nuclear localization promotes cell survival in response to cisplatin

**Figure 3:**
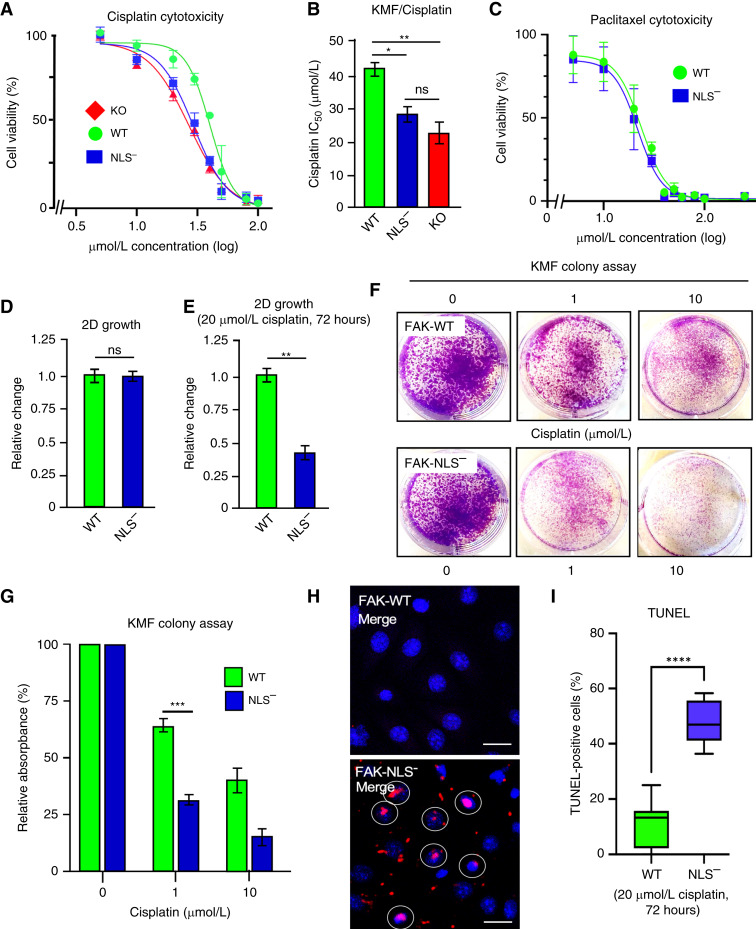
FAK nuclear localization enhances murine ovarian tumor cell survival to cisplatin. **A,** FAK-KO, FAK-WT, and FAK-NLS^−^ KMF cells were evaluated for cisplatin cytotoxicity after 48 hours in culture. Shown is percent cell viability vs. cisplatin concentration (μmol/L, log_10_), and points are means of triplicate samples ± SD (*n* = 3 independent experiments). **B,** Determination of cisplatin IC_50_ values as performed in **A** (*, *P* < 0.05; **, *P* < 0.01). **C,** FAK-WT and FAK-NLS^−^ KMF cells were evaluated for paclitaxel cytotoxicity (μmol/L, log_10_) after 48 hours in culture. **D,** Analysis of KMF-FAK-WT and KMF-FAK-NLS^−^ cells for growth in culture and (**E**) in the presence of 20 μmol/L cisplatin for 72 hours. Values are means ± SD from two independent experiments with triplicate points and FAK-WT values set to 1 (**, *P* < 0.01). **F,** Representative images of crystal violet–stained KMF FAK-WT and FAK-NLS^−^ cell colonies formed in the presence of 0, 1, or 10 μmol/L cisplatin after 10 days. **G,** Quantitation of FAK-WT (green bars) and FAK-NLS^−^ (blue bars) colony formation. Values are means ± SD from three independent experiments with triplicate points (***, *P* < 0.001). **H,** Representative images of TUNEL staining to detect changes in FAK-WT or FAK-NLS^−^ cell DNA fragmentation after cisplatin (20 μmol/L, 48 hours) addition. TUNEL-positive nuclei (identified by DAPI costain) are marked (white circle) in the merged images. Scale bar, 10 μm. **I,** Quantification of FAK-WT (green bars) and FAK-NLS^−^ (blue bars) TUNEL staining. Box and whisker plots show the mean ± SD from three independent experiments (****, *P* < 0.0001). ns, not significant.

To evaluate quantitatively the cellular survival response to cisplatin addition, KMF FAK-KO, FAK-WT, and FAK-NLS^−^ cells were evaluated for viability after 48 hours with increasing concentrations of cisplatin (Fig. 3A). IC_50_ values were determined: FAK-KO, 20.1 ± 4.5 μmol/L; FAK-NLS^−^, 29.3 ± 2.4 μmol/L; and FAK-WT, 40.2 ± 3.9 μmol/L cisplatin, and the difference between FAK-WT to FAK-NLS^−^ and FAK-KO was significant (Fig. 3B). Interestingly, both FAK-WT and FAK-NLS^−^ cells showed equal resistance to paclitaxel after 48 hours (Fig. 3C). Together, these results support the notion that FAK may respond differently to chemotherapy stress as neither cisplatin nor paclitaxel are known to directly bind FAK.

Importantly, KMF FAK-WT and FAK-NLS^−^ cells exhibited equivalent growth in culture (Fig. 3D), with 20 μmol/L cisplatin having strong inhibitory effects on FAK-NLS^−^ cell growth after 72 hours compared with FAK-WT cells (Fig. 3E). As such, KMF colony overgrowth assays showed significantly increased cell growth and survival of FAK-WT compared with FAK-NLS^−^ cells in the presence of 1 or 10 μmol/L cisplatin over 10 days (Fig. 3F and G). At 20 μmol/L cisplatin for 48 hours, FAK-NLS^−^ cells exhibited significantly elevated TUNEL staining compared with FAK-WT cells as a marker associated with cell death (Fig. 3H and I).

**Figure 4:**
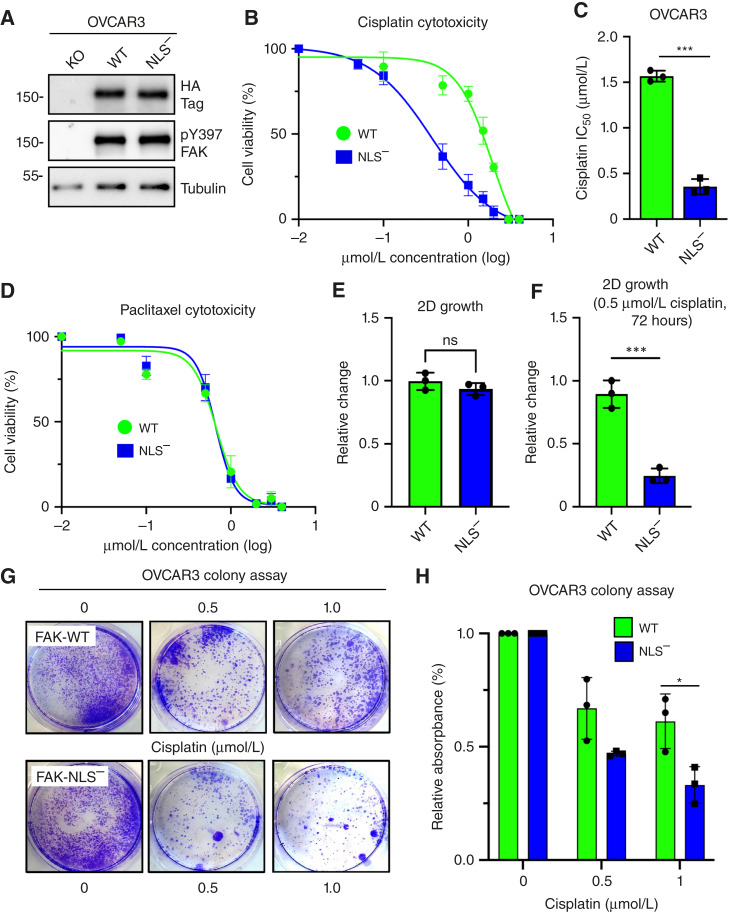
FAK KO and reexpression show that nuclear FAK promotes cisplatin but not paclitaxel resistance in human OVCAR3 cells. **A,** Immunoblots of cultured FAK KO OVCAR3 (clone AB21) and GFP-FAK-WT or FAK-NLS^−^ reconstituted AB21 cells for HA-tag (at FAK C-terminal), FAK pY397, and β-tubulin as a loading control. **B,** FAK-WT, and FAK-NLS^−^ OVCAR3 cells were evaluated for cisplatin cytotoxicity after 48 hours in culture. Shown is percent cell viability vs. cisplatin concentration (μmol/L, log_10_), and points are means of triplicate samples ± SD (*n* = 3 independent experiments). **C,** Determination of IC_50_ values to cisplatin as performed in **B** (***, *P* < 0.001). **D,** FAK-WT and FAK-NLS^−^ OVCAR3 cells were evaluated for paclitaxel cytotoxicity (μmol/L, log_10_) after 48 hours in culture. **E,** Analysis of OVCAR3 FAK-WT and FAK-NLS^−^ cells for growth in culture and (**F**) in the presence of 0.5 μmol/L cisplatin for 72 hours. Values are means ± SD from two independent experiments with triplicate points and FAK-WT values set to 1 (***, *P* < 0.001; ns not significant). **G,** Representative images of crystal violet–stained OVCAR3 FAK-WT and FAK-NLS^−^ cell colonies formed in the presence of 0, 0.5, or 1.0 μmol/L cisplatin after 10 days. **H,** Quantitation of FAK-WT (green bars) and FAK-NLS^−^ (blue bars) colony formation. Values are means ± SD from three independent experiments with triplicate points (*, *P* < 0.05).

To show that FAK nuclear localization is important in promoting human ovarian carcinoma cell survival, GFP-FAK-WT and GFP-FAK-NLS^−^ were stably reexpressed in FAK KO OVCAR3 cells (Fig. 4A). Notably, both FAK-WT and FAK-NLS^−^ were phosphorylated at Y397 in growing cells (Fig. 4A). However, FAK-WT OVCAR3 cells exhibited significantly increased resistance to cisplatin compared with FAK-NLS^−^ OVCAR3 cells (Fig. 4B and C). No differences in sensitivity to paclitaxel or normal growth in cell culture were observed (Fig. 4D and E). In contrast, growth of FAK-NLS^−^ OVCAR3 cells was significantly inhibited by 0.5 μmol/L cisplatin after 72 hours (Fig. 4F) and by 1 μmol/L cisplatin in colony overgrowth assays over 10 days compared with FAK-WT OVCAR3 cells (Fig. 4G and H). Taken together, these results show that nuclear FAK provides a growth and survival advantage in the presence of cisplatin in both murine and human ovarian carcinoma cells.

### FAK nuclear localization supports orthotopic tumor cisplatin resistance

**Figure 5:**
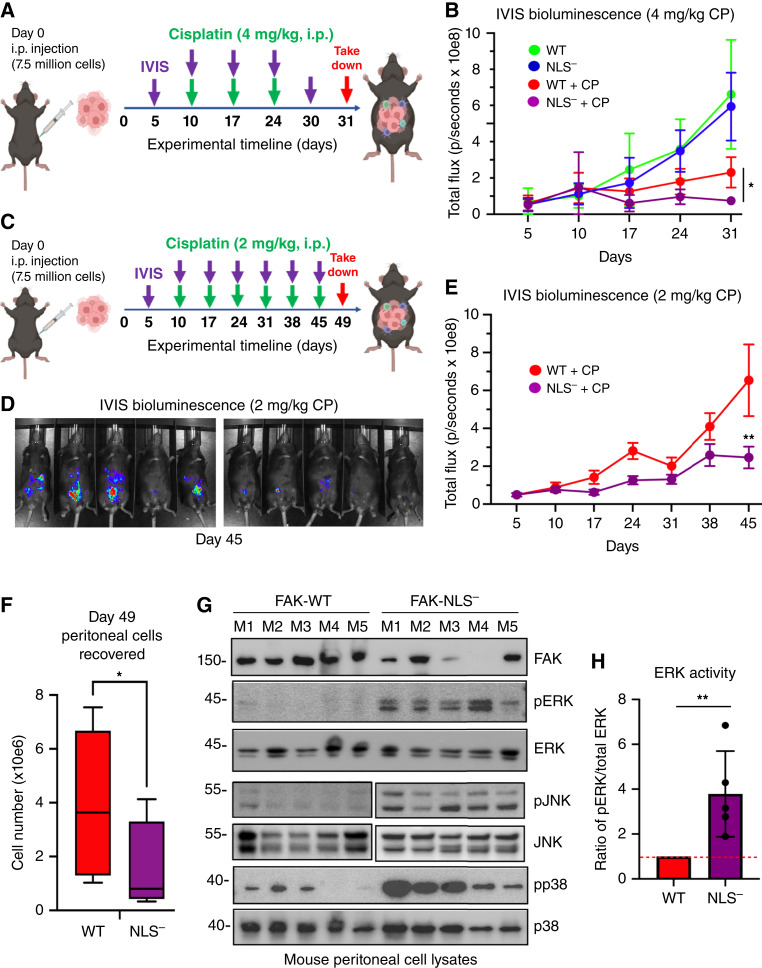
FAK nuclear localization supports KMF tumor cisplatin resistance *in vivo* with effects on downstream signaling. **A,** Experimental schematic: 7.5 million luciferase-expressing KMF FAK-WT or FAK-NLS^−^ cells were intraperitoneally injected into C57Bl6 mice (day 0), and IVIS luciferase imaging at day 5 was used to randomize experimental groups (*n* = 8 each). Mice were administered saline or cisplatin (CP, 4 mg/kg) and imaged on the indicated days. **B,** Graphical presentation of IVIS tumor burden from day 5 to day 31 as expressed as total flux in photons per second. Experimental groups are FAK-WT saline (green), FAK-WT plus CP (red), FAK-NLS^−^ saline (blue), and FAK-NLS^−^ + CP (violet). Values are means ± SD (*n* = 8, *, *P* < 0.05). **C,** Experimental schematic (similar as described in **A**) with decreased and more frequent cisplatin (2 mg/kg) administration over a longer experimental period. **D,** Representative IVIS images of CP-treated FAK-WT and FAK-NLS^−^ tumor-bearing mice on day 45. **E,** Graphical presentation of IVIS tumor burden from day 5 to day 45. Experimental groups are FAK-WT + CP (red) and FAK-NLS^−^ + CP (violet). Values are means ± SD (*n* = 8, **, *P* < 0.01). **F,** Enumeration (trypan blue–negative) of peritoneal cells recovered after euthanasia. Box and whisker plots show the mean (±SD, *, *P* < 0.05) for FAK-WT (red) and FAK-NLS^−^ (violet) tumor-bearing mice. **G,** Protein cell lysates were made from peritoneal collected cells from individual FAK-WT and FAK-NLS^−^ tumor-bearing mice (*n* = 5 each, M1 to M5) and immunoblotted for FAK, β-tubulin, active ERK (pERK), total ERK, active p38 (pp38), and total p38 MAPK. Immunoblotting for active JNK (pJNK) and total JNK were from the same samples but independent gels. **H,** Image quantitation of pERK to total ERK ratio from samples in **G**. Values are means ± SD (*n* = 5 experimental points, **, *P* < 0.01). FAK-WT values set to 1 (dotted line).

To test whether differences in cisplatin resistance *in vitro* extend to tumor growth in mice, KMF FAK-WT and FAK-NLS^−^ cells were labeled with a luciferase reporter and injected into the i.p. space of C57Bl6 mice. After randomization at day 5, tumor burden was indirectly measured by bioluminescent *in situ* imaging, with mice receiving cisplatin (4 mg/kg) or saline (control) injections every 7 days (Fig. 5A and B). Notably, FAK-WT and FAK-NLS^−^ tumors grew equally under control conditions, and 4 mg/kg cisplatin inhibited both FAK-WT and FAK-NLS^−^ after 31 days (Fig. 5B). Cisplatin-treated FAK-NLS^−^ tumors were significantly smaller than FAK-WT tumors, and both were too small to accommodate postexperimental analyses.

To determine whether greater differences in FAK-WT and FAK-NLS^−^ tumor growth may be revealed when cisplatin dosage is reduced (2 mg/kg), a similar mouse tumor experiment was performed (Fig. 5C). Representative IVIS imaging at day 45 showed that cisplatin-treated FAK-WT tumor burden was greater than that of FAK-NLS^−^ tumor-bearing mice (Fig. 5D) and was significantly greater at day 49 (Fig. 5E). Isolation and enumeration of cells collected from a peritoneal wash of tumor-bearing mice at day 49 confirmed significantly more viable cells recovered in FAK-WT versus FAK-NLS^−^ tumor-bearing mice (Fig. 5F). Immunoblotting of peritoneal protein cell lysates from tumor-bearing mice confirmed high levels of GFP-FAK-WT expression (Fig. 5G). In contrast, lower levels of GFP-FAK-NLS^−^ (compared with lysates of FAK-WT tumor-bearing mice) were detected with high levels of active ERK (pERK), active JNK (pJNK), and active p38 (pp38) MAPKs in peritoneal protein lysates of FAK-NLS^−^ tumor-bearing mice (Fig. 5G). ERK activation was significantly increased in peritoneal protein cell lysates of FAK-NLS^−^ compared with GFP-FAK-WT tumor-bearing mice (Fig. 5H). Taken together, these results support the conclusion that nuclear FAK enhances tumor resistance to cisplatin *in vivo* and that cisplatin sensitivity of FAK-NLS^−^ tumors is associated with noncanonical ERK, JNK, and p38 MAPK activation.

### FAK expression, intrinsic activity, and nuclear localization *prevent* cisplatin-stimulated ERK activation

**Figure 6:**
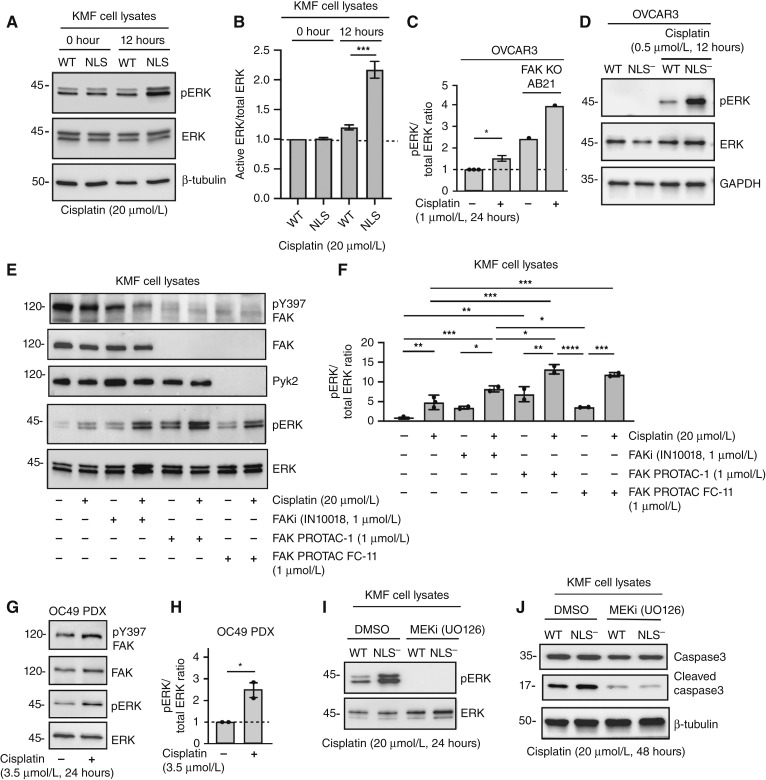
Prevention of FAK expression, activity, or nuclear localization results in elevated cisplatin-stimulated ERK activation. **A,** KMF FAK-WT and FAK-NLS^−^ cells were treated for 0 or 12 hours with cisplatin (20 μmol/L), and cell lysates were immunoblotted for active ERK (pERK), total ERK, and β-tubulin. **B,** Image quantitation of pERK to total ERK ratio from two independent experiments from **A**. Values are means ± SD with control FAK-WT values set to 1 (***, *P* < 0.001). **C,** Image quantitation of pERK to total ERK ratio from parental OVCAR3 and FAK-KO AB21 cells treated with cisplatin (1 μmol/L) for 24 hours. Values are means ± SD from two independent experiments with control OVCAR3 values set to 1 (*, *P* < 0.05). **D,** Representative pERK, total ERK, and GAPDH loading control immunoblotting of OVCAR3 FAK-WT and FAK-NLS^−^ lysates ± cisplatin (0.5 μmol/L, 12 hours). **E,** KMF parental cells were preincubated (48 hours) with FAKi (IN10018, 1 μmol/L), FAK-specific PROTAC (FAK PROTAC-1, 1 μmol/L), and FAK-Pyk2 targeting PROTAC (FAK PROTAC FC-11, 1 μmol/L) followed by with cisplatin addition (20 μmol/L, 24 hours), as indicated. Cell lysates were immunoblotted for pY397 FAK, FAK, and Pyk2 and for active (pERK) and total ERK. **F,** Image quantitation of pERK to total ERK ratio from three independent experiments described in **E**. Values are means ± SD with control set to 1 (*, *P* < 0.05; **, *P* < 0.01; ***, *P* < 0.001; ****, *P* < 0.0001). **G,** Representative immunoblotting of OC49 ovarian PDX cell lysates for pY397 FAK, total FAK, pERK, and total ERK ± cisplatin (3.5 μmol/L, 24 hours). **H,** Image quantitation of pERK to total ERK ratio from two independent experiments described in **G**. Values are means ± SD with control set to 1 (*, *P* < 0.05). **I,** KMF FAK-WT and FAK-NLS^−^ cells were treated 20 μmol/L cisplatin (24 hours) with (DMSO) control or MEK1 inhibitor (U0125, 10 μmol/L) addition. Cell lysates were immunoblotted for active and total ERK. **J,** KMF FAK-WT and FAK-NLS^−^ cells were treated 20 μmol/L cisplatin (48 hours) as above with MEK1 inhibitor and cell lysates blotted for caspase-3, cleaved caspase-3, and β-tubulin. MEKi, MEK inhibitor.

Despite numerous findings of active ERK-promoting tumorigenesis (19), there is also evidence that ERK/MAPK activation can be connected to cisplatin-induced tumor cell death (20, 21). To determine if the observed cisplatin-increased ERK activation in peritoneal protein lysates of FAK-NLS^−^ tumor-bearing mice was a tumor cell–intrinsic response, KMF FAK-WT and KMF FAK-NLS^−^ cells were treated with cisplatin (20 μmol/L for 12 hours) and lysates immunoblotted for total and active ERK (Fig. 6A). Cisplatin-stimulated ERK activation was significantly higher in KMF FAK-NLS^−^ compared with FAK-WT cells (Fig. 6B). In parental human OVCAR3 cells, cisplatin treatment (1 μmol/L for 24 hours) increased the pERK/ERK ratio, and ERK activation was further elevated in FAK-KO OVCAR3 cells (Fig. 6C). As treatment of OVCAR3 FAK-WT and FAK-NLS^−^ reconstituted cells with cisplatin (0.5 μmol/L for 12 hours) resulted in strong ERK activation in FAK-NLS^−^ cell lysates (Fig. 6D), our results support the notion that FAK nuclear localization is important in restraining cisplatin-induced ERK activation in murine and human HGSOC cells.

To determine the role of endogenous FAK expression and intrinsic FAK activity in ERK activation to cisplatin, early-passage KMF cells were treated with a small-molecule FAK-specific inhibitor (FAKi, IN10018) or FAK PROTAC (proteolysis targeting chimera) degraders (FAK PROTAC-1 or FAK FC-11) in the presence or absence of cisplatin (Fig. 6D and E). As anticipated, cisplatin (20 μmol/L for 24 hours) treatment resulted in KMF ERK activation. Notably, FAK inhibition or PROTAC-mediated degradation of FAK protein expression also resulted in significantly elevated basal and cisplatin-stimulated ERK activation compared with controls (Fig. 6D and E). ERK activation was also significantly increased upon cisplatin (3.5 μmol/L for 24 hours) treatment of early-passage human ovarian PDX cells (Fig. 6G and H). To determine if the upstream kinase of ERK (MEK1) was important in regulating cisplatin-stimulated ERK activation, KMF FAK-WT and KMF FAK-NLS^−^ cells were treated with a MEK1 inhibitor (U0126, 10 μmol/L) in the presence of cisplatin for 24 or 48 hours (Fig. 6I and J). FAK-NLS^−^ cell–associated cisplatin-stimulated ERK activation was blocked by MEK1 inhibition and prevented cisplatin-induced caspase-3 cleavage as an indirect marker of cell death. Taken together, our results support the notion that the loss of FAK expression, inhibition of FAK activity, or prevention of FAK nuclear localization result in elevated cisplatin-triggered MEK-ERK activation associated with cell death.

### Nuclear FAK regulation of MKP1 expression supports cisplatin resistance

**Figure 7:**
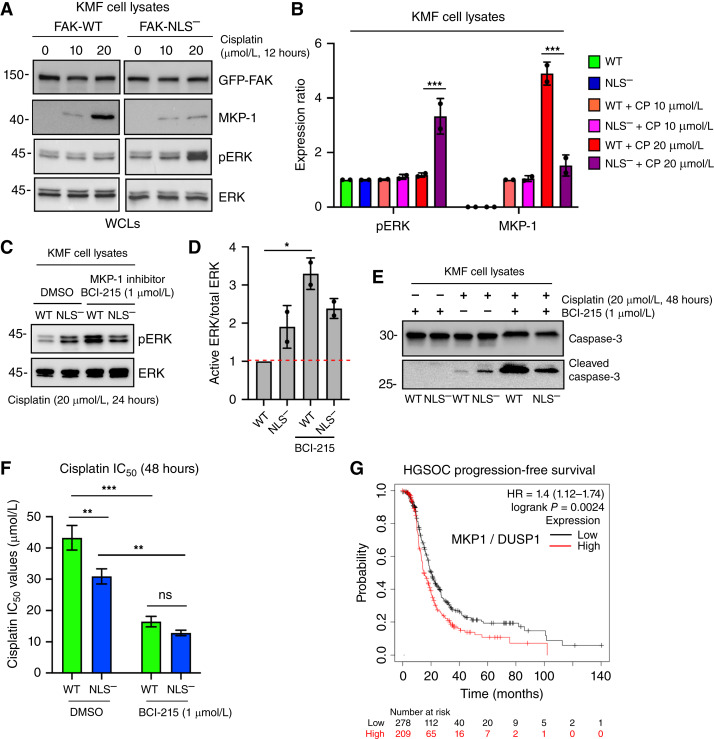
Nuclear FAK regulation of MKP1 expression is associated with cisplatin resistance. **A,** KMF FAK-WT and FAK-NLS^−^ cells were treated with increased concentrations of cisplatin (0, 10, or 20 μmol/L) for 12 hours and protein lysates were immunoblotted for GFP-FAK, MKP1, pERK, and total ERK. **B,** Image quantitation of pERK to total ERK ratio or MKP1 levels (FAK-WT set to 0) from two independent experiments described in **A**. Values are means ± SD (***, *P* < 0.001). **C,** KMF FAK-WT and FAK-NLS^−^ cells were treated 20 μmol/L cisplatin (24 hours) under control (DMSO) or with MKP1 inhibitor (BCI-215, 1 μmol/L) addition. Protein lysates were immunoblotted for pERK and total ERK. **D,** Image quantitation of pERK to total ERK ratio from two independent experiments described in **C**. Values are means ± SD with FAK-WT control set to 1 (*, *P* < 0.05). **E,** KMF FAK-WT and FAK-NLS^−^ cells were treated 20 μmol/L cisplatin (48 hours) as above with MKP1 inhibitor (BCI-215, 1 μmol/L) addition, and cell lysates were immunoblotted for caspase-3 and cleaved caspase-3. **F,** Determination of IC_50_ values to cisplatin KMF FAK-WT (green bars) and FAK-NLS^−^ (blue bars) cells in the presence of MKP1 inhibitor (BCI-215, 1 μmol/L) or DMSO control as determined by alamarBlue cell viability assay. Values are means ± SD from three independent experiments (*, *P* < 0.05; **, *P* < 0.01; ***, *P* < 0.001). **G,** Kaplan–Meier curves from 949 HGSOC patient samples that received adjuvant carbo- or cisplatin-based chemotherapy. Plots show probability of relapse-free survival in months with tumors high (red) or low (black) for MKP1 mRNA (HR = 1.4, *P* = 0.0024). ns, not significant; WCL, whole-cell protein lysate.

To determine potential molecular mechanisms associated with FAK and cisplatin resistance, we noted that previous RNA sequencing results from KMF cells identified MKP1 mRNA as being regulated by FAK expression and activity (28). MKP1 (also known as DUSP1) is a dual-specificity phosphatase known to downregulate ERK, JNK, and p38 MAPK activation (33). As MKP1 expression is stimulated by cisplatin (21) and is associated with HGSOC chemotherapy resistance (24), immunoblotting was used to measure MKP1 protein expression in KMF FAK-WT and FAK-NLS^−^ cells with increasing cisplatin addition (Fig. 7A and B). In lysates from FAK-WT and FAK-NLS^−^ control and 10 μmol/L cisplatin-stimulated cells at 12 hours, only low levels of active ERK and MKP1 levels were detected (Fig. 7B). With 20 μmol/L cisplatin stimulation for 12 hours, MKP1 expression was elevated in FAK-WT but not FAK-NLS^−^ cells (Fig. 7A). Correspondingly, 20 μmol/L cisplatin for 12 hours significantly increased ERK activation in FAK-NLS^−^ but not FAK-WT cells (Fig. 7B). Notably, addition of a small-molecule inhibitor of MKP1 phosphatase activity (BCI-215, 1 μmol/L) resulted in higher levels of ERK activation in cisplatin-stimulated FAK-WT cells compared with control cells (Fig. 7C and D). The combination of MKP1 inhibition and cisplatin stimulation resulted in caspase-3 cleavage in KMF FAK-WT cells (Fig. 7E) and a significant decrease in cisplatin IC_50_ values (Fig. 7F). Finally, with respect to HGSOC patient statistics, Kaplan–Meier analyses show that high MKP1 levels are associated with significantly decreased progression-free survival (Fig. 7G). Taken together, high MKP1 levels are associated with FAK activation and chemotherapy resistance. Moreover, our results support an important role for FAK nuclear accumulation in supporting cisplatin resistance in part by limiting ERK activation via increased MKP1 expression.

## Discussion

Platinum-based chemotherapy is widely used. In HGSOC, carboplatin plus paclitaxel chemotherapy is standard-of-care treatment (2). Nonetheless, most patients develop chemotherapy resistance during initial or subsequent disease recurrence. To sensitize cells to therapy, one approach is to combine cisplatin with drugs that specifically prevent tumor mechanisms of drug resistance. Because FAK is expression is elevated in most solid tumors, and FAK is activated by a variety of cell stimuli—mechanical, growth factor, extracellular matrix, cytokines, and cellular stress—it is an attractive target supporting drug resistance (34). FAK inhibition studies have revealed multiple potential tumor vulnerabilities and potential paths for interception. Precision inhibitors to blocking FAK activity have been developed, and these drugs are orally available and tolerated with few adverse events (16).

Although FAK activation in response to platinum agents has been shown in prior studies (28), the intense localization of FAK to the nucleus after subcytotoxic cisplatin addition to tumor cells was unexpected, and reminiscent of the nuclear shuttling of other integrin-activated tyrosine kinases, such as Abl (35). Herein, we show that active FAK staining increases in the nucleus of HGSOC patient tumors after neoadjuvant carboplatin–paclitaxel chemotherapy. This response was tumor cell–intrinsic, as increased FAK tyrosine phosphorylation occurred within 1 hour, whereas nuclear FAK accumulation was observed within 12 hours after subcytotoxic cisplatin treatment of ovarian tumor cells. The timing of FAK activation after cisplatin stimulus is consistent with studies that document FAK phosphorylation occurring in the cell periphery. Accordingly, mutation of FAK FERM NLS targeting did not limit cisplatin-induced FAK Y397 phosphorylation but did prevent cisplatin-induced FAK nuclear accumulation. Although nuclear FAK can associate with chromatin and transcription factors regulating gene expression (12), the mechanistic role of FAK in the nucleus after cisplatin treatment remains under investigation. However, *in vitro* and *in vivo*, we found that nuclear FAK localization was associated with increased tumor cell survival in response to cisplatin.

**Figure 8:**
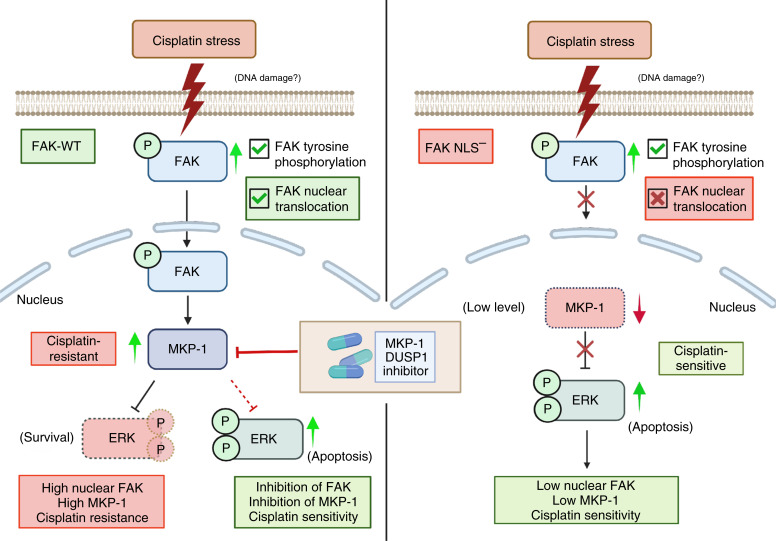
Model of cisplatin-stimulated nuclear FAK protection against ERK-associated cell death in ovarian cancer. Left, Patient tumors surviving neoadjuvant chemotherapy exhibit a high level of active FAK staining in the cell nucleus. This is a tumor cell–intrinsic response, as subcytotoxic cisplatin treatment was associated with FAK nuclear accumulation in human and mouse ovarian tumor cells. Cisplatin stress also increased MKP1 tyrosine phosphatase expression that limits phosphorylation and activity of ERK. Inhibition of MKP1 results in elevated cisplatin-stimulated ERK activity associated with cell death. FAK inhibition also results in increased ERK activity, and FAK inhibition potentiated cisplatin-induced cell death (28). Right, Mutational inactivation of FAK nuclear targeting (FAK-NLS^−^) does not prevent basal or cisplatin-stimulated FAK-NLS^−^ phosphorylation at Y397. FAK-NLS^−^ does not accumulate in the nucleus upon cisplatin addition to cells. FAK-NLS^−^ cells exhibit sensitivity to cisplatin cytotoxicity, reduced MKP1 expression, and elevated ERK activation. Our results support the notion that nuclear FAK supports MKP1 expression and that combinatorial small-molecule targeting of FAK kinase and MKP1 phosphatase activities may function as an effective therapy combination with cisplatin for HGSOC. Created in BioRender (RRID: SCR_018361). Schlaepfer, D. (2024) BioRender.com/n24i207.

FAK has previously been shown to activate the ERK/MAPK pathway following integrin ligation, and this can sustain ERK activation and breast cancer tumor progression (18, 36). In contrast, our results support a model (Fig. 8) whereby nuclear FAK limits cisplatin-induced ERK activation in HGSOC. This occurs in part by supporting cisplatin-stimulated MKP1 phosphatase expression. MKP1 expression is elevated in HGSOC, MKP1 is associated with cisplatin resistance, and small-molecule inhibitors of MKP1 phosphatase activity inhibit HGSOC cell proliferation and promote cell death (22–24). These results suggest that nuclear FAK regulation of MKP1 expression is associated with cisplatin resistance (Fig. 8). We hypothesize that cisplatin-induced FAK activation, a cellular intrinsic adaptive resistance response (37), is maintained over several hours. FAK nuclear accumulation supports MKP1 expression that limits ERK phosphorylation and, as such, ERK activity. Indeed, others have previously demonstrated that among HGSOC, elevated ERK activity following cisplatin exposure was associated with cell death (20). A role for FAK is evident, as different approaches to inhibit FAK by PROTAC-mediated FAK degradation resulted in elevated basal and cisplatin-induced ERK activation. As MKP-1 mRNA expression was elevated by intrinsic FAK activity (28), we speculate that a kinase-dependent role for nuclear FAK may regulate MKP1 expression. Interestingly, extended treatment of uveal melanoma cell lines with a chemically different small-molecule FAKi (Verastem, VS-4718) also resulted in higher levels of basal ERK activity (38), suggesting that our observed responses with murine ovarian and HGSOC cells may be a more general tumor cell response to FAK inhibition.

Given this, it is perhaps not surprising that FAK-NLS^−^ cells exhibit sensitivity to cisplatin cytotoxicity, have reduced MKP1 expression, and have elevated ERK activation. Although MKP1 small-molecule inhibitors remain in preclinical development, and BCI-215 inhibited HGSOC cell proliferation *in vitro* and in mice (24), many of the pharmacoproperties of BCI-215 remain unknown. Our results support the notion that nuclear FAK supports MKP1 expression and that combinatorial small-molecule targeting of FAK kinase and MKP1 phosphatase activities may function as an effective therapy combination with cisplatin for HGSOC.

Lastly, we speculate that FAK signaling in HGSOC tumors may be molecularly different than the proposed role of FAK in LGSOC (39). Notably, LGSOC tumors frequently exhibit activation of the RAS/RAF/MEK/ERK axis, display high level of ERK/MAPK activity, and accordingly exhibit MEK inhibitor sensitivity (40). Clinical trials are currently testing the combination of a Raf-MEK inhibitor (avutometinib) with a FAKi (defactinib) for KRAS-mutated NSCLC (25) and LGSOC (26). Similar to chemotherapy-treated colon cancer (37), increased FAK tyrosine phosphorylation is detected in avutometinib-treated KRAS-mutated NSCLC cells (25) wherein FAK activation in this context may represent an adaptive or intrinsic cell resistance mechanism. Our findings support the notion that FAK inhibition is intertwined with ERK activation HGSOC, whereas in LGSOC or NSCLC, FAK inhibition works in parallel with ERK inhibition. Together, our results support the notion that FAK nuclear localization and either positive or negative signaling effects on ERK activity is likely tumor type– and chemotherapy-dependent.
